# Comparative proteomics of two *Mycoplasma hyopneumoniae* strains and *Mycoplasma flocculare* identified potential porcine enzootic pneumonia determinants

**DOI:** 10.1080/21505594.2018.1499379

**Published:** 2018-08-12

**Authors:** Jéssica Andrade Paes, Lais Del Prá Netto Machado, Fernanda Munhoz dos Anjos Leal, Sofia Nóbrega De Moraes, Hercules Moura, John R. Barr, Henrique Bunselmeyer Ferreira

**Affiliations:** a Laboratório de Genômica Estrutural e Funcional, Centro de Biotecnologia, Universidade Federal do Rio Grande do Sul, Porto Alegre, Brazil; b Biological Mass Spectrometry Laboratory, Clinical Chemistry Branch, Division of Laboratory Sciences, National Center for Environmental Health, Centers for Disease Control and Prevention, Atlanta, GA, USA

**Keywords:** Comparative proteomics, swine respiratory pathogens, mycoplasma, subcellular fractions, virulence factors, bacterium-host interactions

## Abstract

*Mycoplasma hyopneumoniae* and *Mycoplasma flocculare* are genetically similar bacteria, which coinhabit the porcine respiratory tract. These mycoplasmas share most of the known virulence factors, but, while *M. hyopneumoniae* causes porcine enzootic pneumonia (PEP), *M. flocculare* is a commensal species. To identify potential PEP determinants and provide novel insights on mycoplasma-host interactions, the whole cell proteomes of two *M. hyopneumoniae* strains, one pathogenic (7448) and other non-pathogenic (J), and *M. flocculare* were compared. A cell fractioning approach combined with mass spectrometry (LC-MS/MS) proteomics was used to analyze cytoplasmic and surface-enriched protein fractions. Average detection of ~ 50% of the predicted proteomes of *M. hyopneumoniae* 7448 and J, and *M. flocculare* was achieved. Many of the identified proteins were differentially represented in *M. hyopneumoniae* 7448 in comparison to *M. hyopneumoniae* J and *M. flocculare*, including potential PEP determinants, such as adhesins, proteases, and redox-balancing proteins, among others. The LC-MS/MS data also provided experimental validation for several genes previously regarded as hypothetical for all analyzed mycoplasmas, including some coding for proteins bearing virulence-related functional domains. The comprehensive proteome profiling of two *M. hyopneumoniae* strains and *M. flocculare* provided tens of novel candidates to PEP determinants or virulence factors, beyond those classically described.

## Introduction

The identification and characterization of virulence factors is of upmost relevance to discover new targets for the development of diagnostic methods, therapeutic drugs, and vaccines [1]. However, the multifactorial nature of pathogenicity poses difficulties to identify disease-related proteins and mechanisms in pathogenic species. Comparisons between virulent and avirulent strains of a pathogenic species and/or two closely-related species that coinhabit the same host species, being one pathogenic and the other a commensal, are expected to provide valuable information on determinants of pathogenic/commensal ways of life.

Among the mycoplasmas that coinhabit the swine respiratory tract, there are two interesting species for comparative studies: the pathogenic *Mycoplasma hyopneumoniae* and the commensal *Mycoplasma flocculare* [2]. *M. hyopneumoniae* adheres to the host respiratory epithelium and causes the porcine enzootic pneumonia (PEP). *M. flocculare* also adheres to porcine respiratory epithelium and can be isolated from normal and pneumonic lungs. This species is usually regarded as non-pathogenic [3,4], although it is considered by some authors an opportunistic pneumonic pathogen in coinfections with *M. hyopneumoniae* [5]. Despite the pathogenic nature of *M. hyopneumoniae*, there are some strains that vary in their virulence levels, or even are avirulent, such as *M. hyopneumoniae* J, which has reduced adhesion capacity to porcine cilia [6]. Comparisons between the genomes of *M. hyopneumoniae* pathogenic and non-pathogenic strains (7448 and J, respectively) revealed no extensive genomic differences [7]. Moreover, previous comparative phylogenetic and phylogenomic studies provided evidences of the close relationship of *M. hyopneumoniae* and *M. flocculare* [7–9], which share most of the known virulence-related genes [10]. The differences between *M. hyopneumoniae* and *M. flocculare* include the absence, in *M. flocculare*, of the *glpO* gene, related to *M. hyopneumoniae* hydrogen peroxide generation and cytotoxicity [11,12], and differential domains between orthologs from the P97 family of adhesins and from other surface proteins [13]. However, 90% of *M. flocculare* predicted surface proteins are shared with *M. hyopneumoniae* [9], and the observed genomic differences between *M. hyopneumoniae* strains, and between *M. hyopneumoniae* and *M. flocculare* so far do not fully explain their differential phenotypes of virulence/pathogenicity.

Differential expression of ortholog genes may also contribute to differences in pathogenicity or virulence level between *M. hyopneumoniae* strains or between *M. hyopneumoniae* and *M. flocculare*. However, previous comparative transcriptomic studies between *M. hyopneumoniae* and *M. flocculare* [14] failed to find differences in the relative transcription levels for most genes. On the other hand, pioneer proteomic studies, have provided evidences of differential protein abundance and post-translational processing between *M. hyopneumoniae* pathogenic (7448 and 7422) and non-pathogenic (J) strains [15]. Moreover, a recent comparative proteomics study between *M. hyopneumoniae* and *M. flocculare* secreted proteins revealed several virulence-related differences between these mycoplasma species [16]. This study showed that the *M. hyopneumoniae* secretome included several virulence-related proteins, like adhesins, transporters, nucleases and uncharacterized proteins bearing virulence-related functional domains, not found in the *M. flocculare*, secretome. Overall, these previous studies indicate the necessity of further and more comprehensive comparative proteomic studies, to deeply investigate possible pathogenicity or virulence-related differences at the protein level.

Here, the whole cell proteomes of *M. hyopneumoniae* strains 7448 (pathogenic) and J (non-pathogenic), and *M. flocculare* were compared by a mass spectrometry (MS)-based approach to identify differences in protein abundance associated with pathogenicity or virulence. Mycoplasma cells were fractioned into cytoplasmic- and surface-enriched protein fractions and their protein contents were analyzed by high-resolution and high-sensitivity MS. Several significant differences among *M. hyopneumoniae* strains and *M. flocculare* proteomes were depicted, and their biological significance for mycoplasma-host interactions, for virulence and PEP determination are discussed.

## Results

### MS-based identification of proteins from M. hyopneumoniae 7448 and J, and M. flocculare

**Figure 1.**
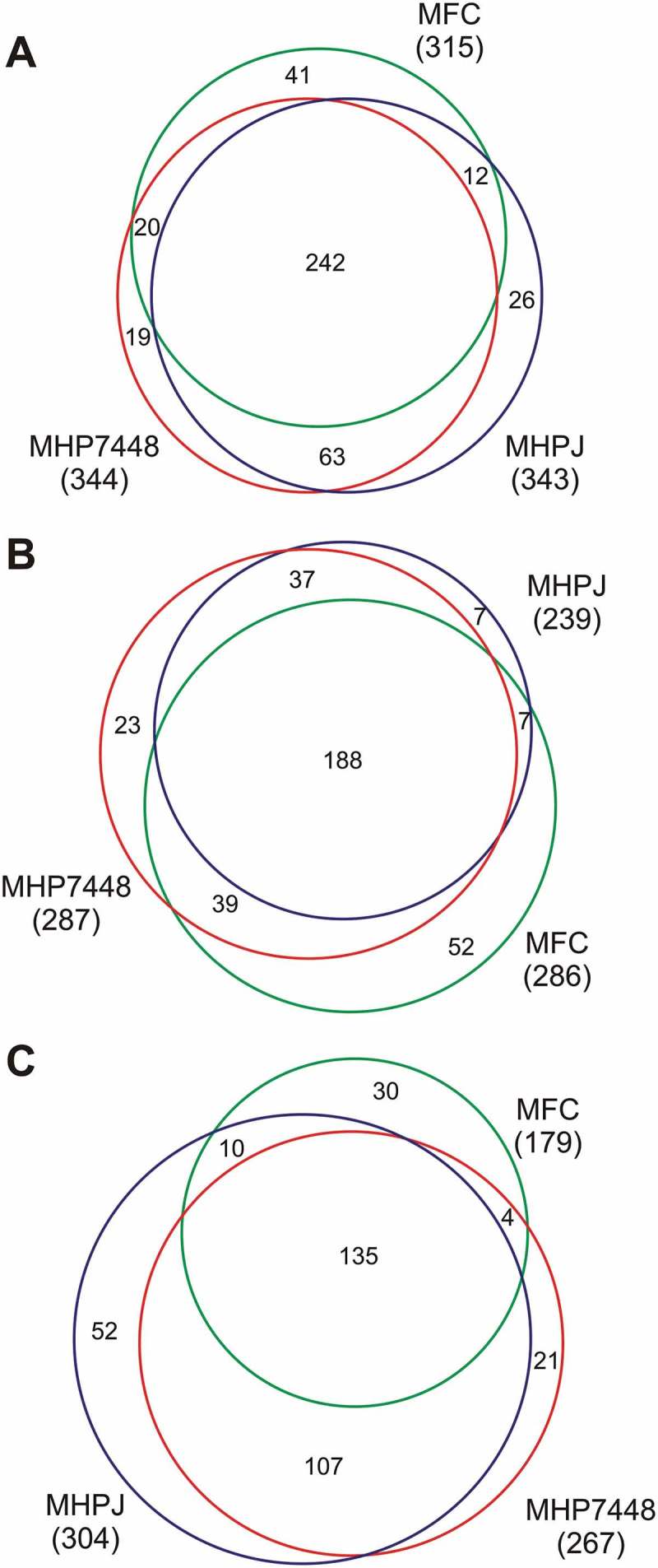
Overview of proteins identified in *M. hyopneumoniae* 7448 (MHP7448) and J (MHPJ), and *M. flocculare* (MFC) samples. (A) Total proteins. (B) Proteins detected in soluble fractions. (C) Proteins detected in insoluble fractions. Overall numbers of proteins identified for each sample between parentheses. Numbers of proteins exclusively detected in each sample or shared between them are indicated within the Venn diagrams.

LC-MS/MS analyzes of proteins from soluble and insoluble fractions identified overall totals of 344 out of 695 (~ 50%), 343 out of 672 (~ 51%), and 315 out of 581(~ 54%) protein species from *M. hyopneumoniae* 7448, *M. hyopneumoniae* J, and *M. flocculare*, respectively. Detailed peptide and protein identification data are presented in Supplementary Tables 1 and 2, respectively. Around 70% (242) of the detected proteins were shared between *M. hyopneumoniae* 7448, J, and *M. flocculare* (Figure 1(A), Supplementary Table 3A). The average peptide coverage for both *M. hyopneumoniae* and *M. flocculare* identified proteins was ~ 40% in soluble fraction and ~ 20% in insoluble fraction. The calculated zero false discovery rates (FDR) for the proteins and peptides of all samples validated all MS/MS results.

Considering the detected proteins in soluble fractions, 287, 239, and 286 proteins were identified in *M. hyopneumoniae* 7448, *M. hyopneumoniae* J, and *M. flocculare* samples, respectively. Most (188) of the identified proteins in this fraction type (considering ortholog ones) were shared between the two *M. hyopneumoniae* strains, and *M. flocculare* (Figure 1(B), Supplementary Table 3B). Considering the number of detected proteins in insoluble fractions, 267 proteins were identified for *M. hyopneumoniae* 7448, 304 proteins, for *M. hyopneumoniae* J, and 179 proteins, for *M. flocculare*. Most (135) proteins identified in these fractions are shared among all analyzed mycoplasmas (Figure 1(C), Supplementary Table 3C).

Overall, these preliminary comparisons indicated qualitative differences among the proteomes of *M. hyopneumoniae* 7448 and J, and *M. flocculare*. Some of these differences between pathogenic and non-pathogenic mycoplasmas may be associated with pathogenicity.

### Enrichment of surface-related proteins in insoluble extracts of *M. hyopneumoniae* 7448 and J, and M. flocculare

The identified protein repertoires from soluble and insoluble protein extracts were compared to confirm the enrichment of the insoluble fraction with surface proteins. *In silico* subcellular localization predictions for proteins identified in insoluble and soluble extracts are detailed in Supplementary Table 4. Higher numbers of proteins predicted as surface proteins were identified in the insoluble fractions of *M. hyopneumoniae* strains 7448 (111; 41.6%) and J (116; 38.2%), and for *M. flocculare* (84; 46.9%) in comparison to those identified in the corresponding soluble extracts (~ 25%). Conversely, most proteins identified in the soluble fractions of *M. hyopneumoniae* 7448 (168; 58.5%), *M. hyopneumoniae* J (144; 60.3%) and *M. flocculare* (161; 56.3%) were predicted as cytoplasmic proteins. Overall, these preliminary results showed qualitative differences between extracts that demonstrate the enrichment of surface proteins in insoluble extracts of *M. hyopneumoniae* (7448 and J) and *M. flocculare*.

**Figure 2.**
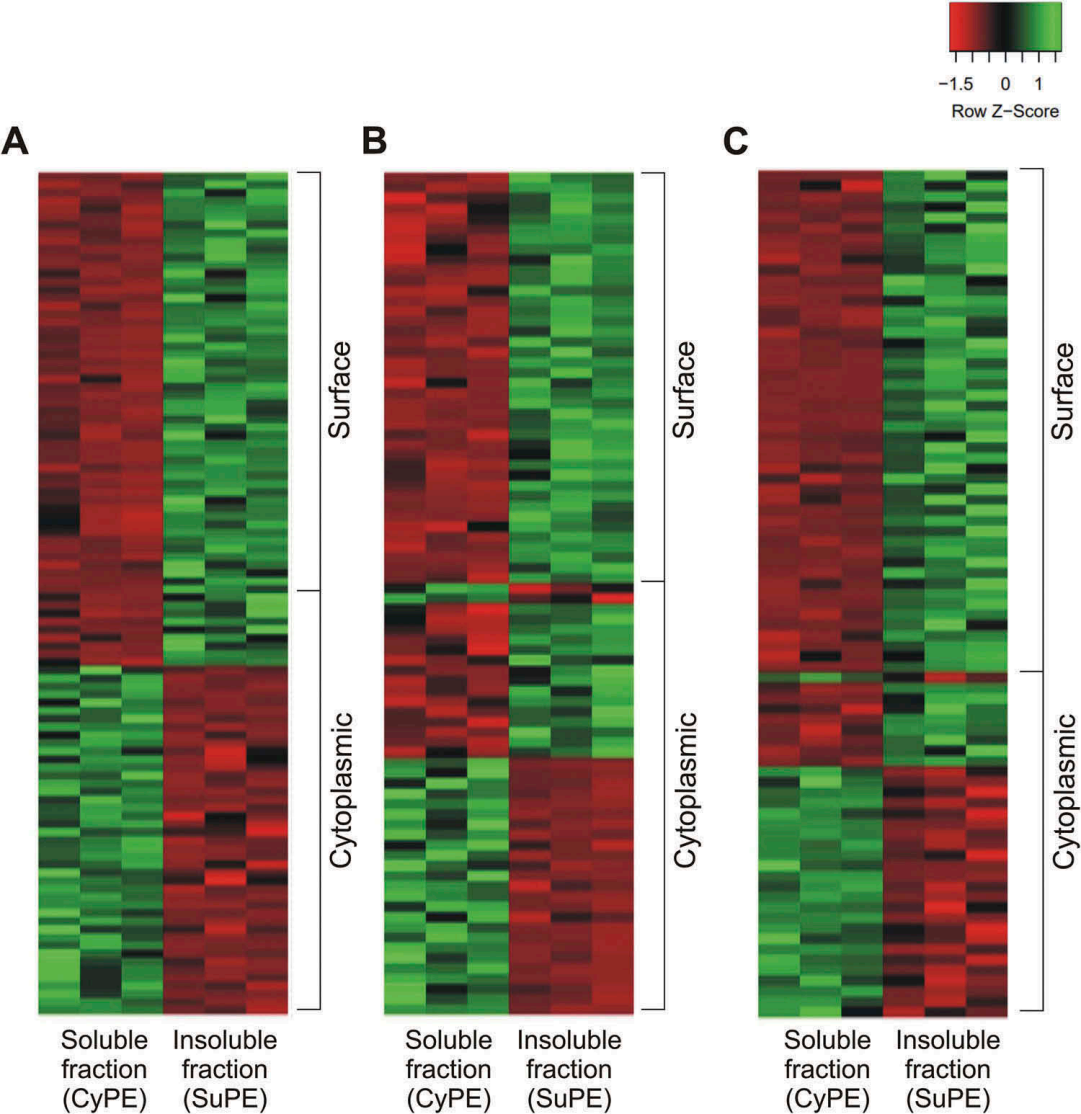
Heatmaps showing the enrichment of surface-related proteins in insoluble extracts of (A) *M. hyopneumoniae* 7448, (B) *M. hyopneumoniae* J, and (C) *M. flocculare*. In each heat map, all shared proteins showing statistically significant abundances (*p* < 0.05) between soluble (CyPE) and insoluble (SuPE) fractions are represented (red, low abundance; green, high abundance). Distribution of surface and cytoplasmic-predicted proteins are indicated on the right. NSAF values, converted in Z-scores, were used to quantify relative differences in protein abundance, and the *t*-test was applied to determine statistically significant differences between shared proteins.

Additionally, to verify the quantitative enrichment of surface proteins in insoluble protein extracts, the differential abundance of shared proteins between soluble and insoluble fractions were assessed using NSAF values. For *M. hyopneumoniae* 7448, 125 out of 210 proteins (59%) were differentially abundant between these fractions, while 93 out of 200 (46%) and 89 out of 150 (59%) were differentially abundant for *M. hyopneumoniae* J, and for *M. flocculare*, respectively (Supplementary Table 4). Among overrepresented proteins, there was an evident enrichment of surface-predicted proteins in insoluble fractions for all analyzed mycoplasmas, while cytoplasmic proteins were more abundant in soluble fractions (Figure 2).

Overall, the repertoires of differential proteins demonstrated the clear enrichments of surface proteins and cytoplasmic proteins in the analyzed insoluble and soluble fractions, respectively. Therefore, from now on these mycoplasma soluble and insoluble fractions will be treated as cytoplasmic-enriched protein extracts (CyPE) and surface-enriched protein extracts (SuPE), respectively.

### Differences between the whole-cell protein contents of M. hyopneumoniae 7448 and J, and M. flocculare

The whole-cell proteome (including proteins detected in both CyPE and SuPE) of *M. hyopneumoniae* 7448 was qualitatively and quantitatively analyzed and compared to those from *M. hyopneumoniae* J and *M. flocculare*. Qualitative comparisons were based on presence/absence of detected ortholog proteins, while quantitative comparisons were performed between ortholog proteins shared between *M. hyopneumoniae* 7448 and J, and between *M. hyopneumoniae* 7448 and *M. flocculare*.

In the whole-cell proteomes, 39 and 82 proteins were exclusively detected in *M. hyopneumoniae* 7448 samples in comparison to *M. hyopneumoniae* J and *M. flocculare*, respectively (see Figure 1). Separately analyzing the CyPEs, 62 and 60 proteins were found exclusively in *M. hyopneumoniae* 7448 samples in comparison to *M. hyopneumoniae* J and *M. flocculare*, respectively. On the other hand, 25 and 128 proteins were *M. hyopneumoniae* 7448-exclusive in comparison to *M. hyopneumoniae* J and *M. flocculare*, respectively, considering only SuPE samples.

Quantitative analyzes were performed with shared proteins between samples based on emPAI values (Supplementary Table 5). *M. hyopneumoniae* 7448 and J strains shared 305 proteins, while *M. hyopneumoniae* 7448 and *M. flocculare* shared 262. Among the proteins shared between *M. hyopneumoniae* 7448 and J strains, 78 proteins were differentially abundant. In comparison to *M. hyopneumoniae* J, 25 CyPE proteins and 18 SuPE proteins were overrepresented in *M. hyopneumoniae* 7448 samples. The differences in abundance ranged from 1.5 to 7.3-fold. Only some SuPE proteins (35 proteins) were underrepresented in *M. hyopneumoniae* 7448 samples in comparison to the J strain.

Quantitative comparisons between *M. hyopneumoniae* 7448 to *M. flocculare* found 79 proteins differentially abundant. Twenty CyPE and 44 SuPE proteins were overrepresented in *M. hyopneumoniae* 7448 samples, with differences in abundance ranging from 1.7 to 63-fold. Nine CyPE and 6 SuPE proteins were underrepresented in *M. hyopneumoniae* 7448 samples in this comparison. Among these differentially abundant proteins from *M. hyopneumoniae* 7448 and *M. flocculare*, only 9 presented significant abundance differences in both CyPE and SuPE. Of these proteins seven were overrepresented in both protein extracts of *M. hyopneumoniae* 7448. The other 2 represent cases of differential enrichment between the subcellular fractions in these two species. An aminopeptidase was overrepresented in the CyPE and underrepresented in the SuPE, in *M. hyopneumoniae* 7448, and vice-versa, in *M. flocculare*. Conversely, an uncharacterized protein (MHP7448_0356), was underrepresented in the CyPE and overrepresented in the SuPE, in *M. hyopneumoniae* 7448, and vice-versa, in *M. flocculare*.

Overall, these results showed important qualitative and quantitative differences between *M. hyopneumoniae* 7448 and J strains, between *M. hyopneumoniae* 7448 and *M. flocculare* regarding whole-cell proteomes. These differences can be associated with the differential pathogenic and non-pathogenic natures of these mycoplasmas and may point out some potential PEP determinants as described in the next sections.

### Potential PEP determinants differentially represented in M. hyopneumoniae 7448

**Figure 3.**
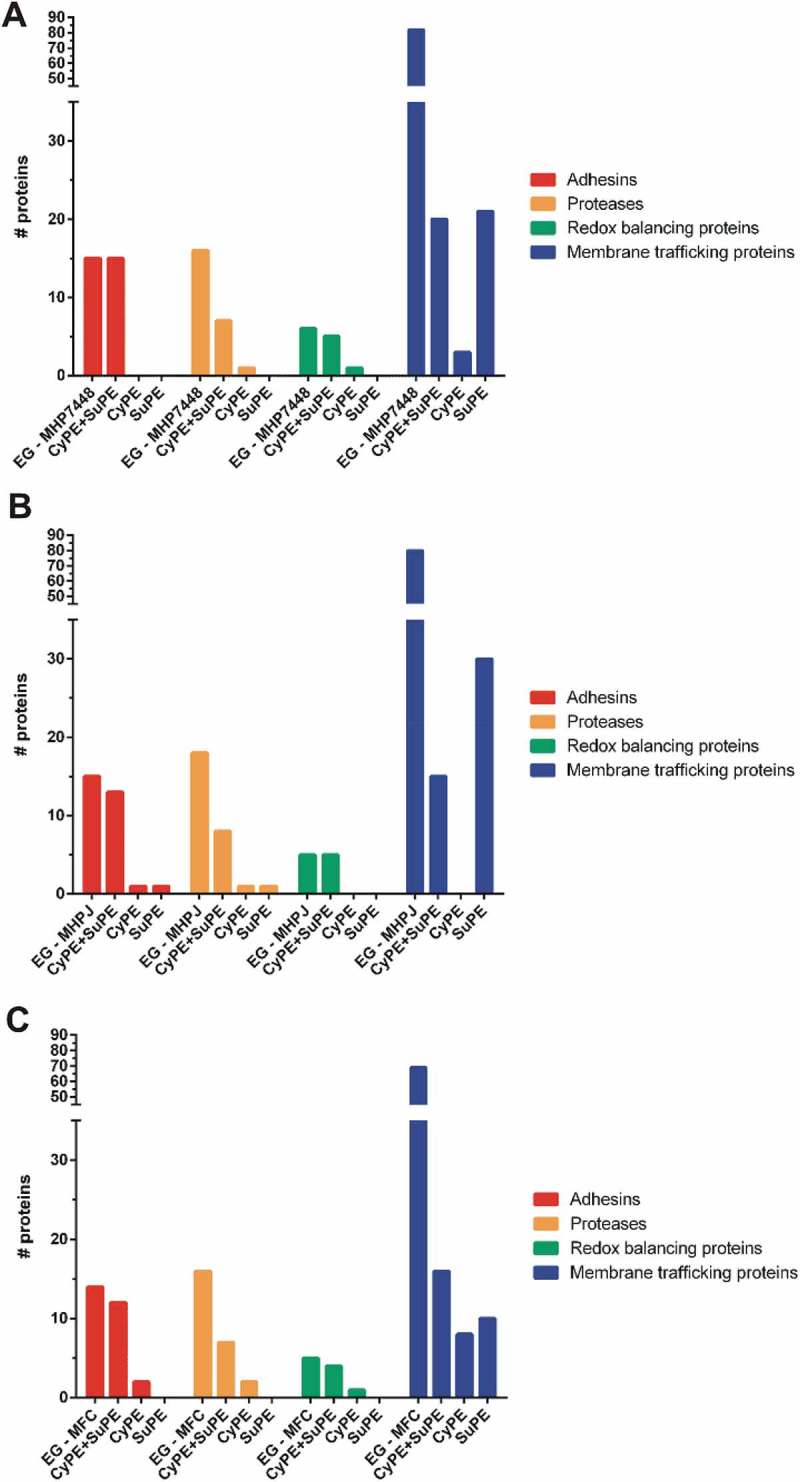
Qualitative differences in potential PEP determinants detected in CyPE and/or SuPE of (A) *M. hyopneumoniae* 7448, (B) *M. hyopneumoniae* J, and (C) *M. flocculare.* Bar colors represent the different classes of potential PEP determinant, as indicated. CyPE+SuPE, proteins detected in both CyPE and SuPE samples; CyPE, proteins detected only in CyPE samples; SuPE, proteins detected only in SuPE samples. A bar representing the overall number of genes encoding each class of PEP determinants in the corresponding mycoplasma genome was included for reference (EG).

Differential proteins between *M. hyopneumoniae* 7448 and its non-pathogenic counterparts were assumed to be potential PEP determinants. This assumption was validated by the fact that, among these differential proteins, there were many virulence-related proteins previously described in the literature, like adhesins, proteases, redox balancing protein, and membrane transporters. The observed qualitative differences are graphically represented in Figure 3, and quantitative differences are presented in Table 1.

**Table 1.** Potential virulence-related proteins overrepresented (*p* < 0.05 and FC > 1.5) in *M. hyopneumoniae* 7448 samples. Association with virulence was based on the cited references.

		Fold-changes^(1)^				
		CyPE^(2)^		SuPE^(2)^		
NCBI accession number	Protein name	MHP7448/MHPJ	MHP7448/MFC	MHP7448/MHPJ	MHP7448/MFC	Reference
MHP7448_0210	ABC transporter ATP-binding protein	2.93	3.13	-	-	[34]
MHP7448_0314	ABC transporter ATP-binding protein	-	2.67	2.35	2.42	[34]
MHP7448_0315	ABC transporter ATP-binding protein	-	-	1.84	-	[34]
MHP7448_0452	ABC transporter ATP-binding protein	-	-	-	3.04	[34]
MHP7448_0129	Aminopeptidase	-	12.75	-	-	[10]
MHP7448_0051	ATP synthase subunit alpha	2.96	-	-	2.28	[70]
MHP7448_0101	ATP-dependent protease binding protein	-	-	-	4.77	[10]
MHP7448_0068	Chaperone protein DnaJ	-	-	-	5.20	[10]
MHP7448_0507	Dihydrolipoyl dehydrogenase	-	-	-	2.61	[41]
MHP7448_0075	Elongation factor G	-	-	-	3.34	[71]
MHP7448_0056	Elongation factor Ts	-	-	-	10.00	[71]
MHP7448_0523	Elongation factor Tu	-	-	-	2.61	[72]
MHP7448_0263	Energy-coupling factor transporter ATP-binding protein EcfA1	-	5.76			[73]
MHP7448_0464	Leucyl aminopeptidase	-	-	2.09	3.95	[10]
MHP7448_0133	Lipase-esterase	-	13.40	-	-	[74]
MHP7448_0137	L-lactate dehydrogenase	-	62.92	-	-	[36]
MHP7448_0524	Lon protease (ATP-dependent protease La)	-	-	-	3.17	[10]
MHP7448_0173	Methionine aminopeptidase	-	3.86	-	-	[10]
MHP7448_0082	NADH oxidase	-	3.90	-	2.48	[75]
MHP7448_0521	Oligoendopeptidase F	-	-	1.59	3.74	[27]
MHP7448_0501	Oligopeptide ABC transporter ATP-binding protein	-	-	-	4.32	[34]
MHP7448_0360	p37-like ABC transporter substrate-binding lipoprotein	-	-	4.24	-	[34]
MHP7448_0272	p97-like protein	2.50	-	-	-	[10]
MHP7448_0161	Phosphopentomutase	3.28	-	-	-	[42]
MHP7448_0656	Prolipoprotein p65	-	4.67	-	2.67	[10]
MHP7448_0376	PTS system ascorbate-specific transporter subunit IIC	-	-	1.58	-	[76]
MHP7448_0375	PTS system enzyme IIB component	3.61	-			[76]
MHP7448_0005	Putative MgpA-like protein	2.84	-	-	-	[10]
MHP7448_0116	Pyruvate dehydrogenase	-	16.00	-	4.35	[41]
MHP7448_0115	Pyruvate dehydrogenase E1-alpha subunit	-	2.61	-	12.91	[41]
MHP7448_0037	Ribonuclease R	3.60	3.03	-	-	[10]
MHP7448_0096	Thiol peroxidase	5.63	-	-	-	[10]
MHP7448_0384	Thioredoxin	-	-	1.55	-	[10]
MHP7448_0098	Thioredoxin reductase	-	-	-	2.81	[10]

^(1)^ Fold changes were based on emPAI quantitative values of MHP7448 divided by those of MHPJ or MFC.

^(2)^ Dashes means that the determined *M. hyopneumoniae* 7448 protein were not differentially abundant in CyPE and/or SuPE and, in comparison to *M. hyopneumoniae* J and/or *M. flocculare*

Qualitative comparisons revealed that most of the differential proteins were detected in both CyPE and SuPE samples for all analyzed mycoplasmas (see Supplementary Table 2). However, some proteins were exclusively detected in only one subcellular fraction, as follows. Methionine aminopeptidase was exclusively found in the CyPE from *M. hyopneumoniae* 7448 (MHP7448_0173), *M. hyopneumoniae* J (MHJ_0169) and, *M. flocculare* (MFC_0210). The XAA-PRO aminopeptidase was exclusively detected in the CyPE in *M. flocculare*, while in both *M. hyopneumoniae* strains it was detected in both CyPE and SuPE. The neutrophil activating factor, which is involved in oxidative stress, was exclusively detected in *M. hyopneumoniae* 7448 CyPE samples (MHP7448_0457). Most of the detected membrane transporters protein species were found only in SuPE or in both CyPE and SuPE samples from all analyzed mycoplasmas. As expected, those membrane transporters shared between CyPE and SuPE samples were mostly enriched in the SuPE samples (see Supplementary Table 4).

Considering the proteins detected in both CyPE and SuPE, several quantitative differences were observed involving the *M. hyopneumoniae* 7448 adhesin repertoire in comparison to those of the non-pathogenic counterparts. In comparison to *M. hyopneumoniae* J, the P97-like (MHP7448_0272) and MgpA-like (MHP7448_0005) adhesins were overrepresented in *M. hyopneumoniae* 7448 CyPE. On the other hand, the P95 (MHP7448_0099) and P102-copy 1 (MHP7448_0199) adhesins, were underrepresented in *M. hyopneumoniae* 7448 SuPE. Comparisons between *M. hyopneumoniae* 7448 and *M. flocculare* adhesins, showed that the P65 adhesin (MHP7448_0656) was overrepresented in *M. hyopneumoniae* 7448 in both CyPE and SuPE. Interestingly, in *M. flocculare*, the P95 (MFC_00492) and P60-like (MFC_01236) adhesins, in CyPE, and the P97 copy-2 adhesin (MFC_00472), in SuPE, were overrepresented in comparison to *M. hyopneumoniae* 7448.

Proteases are often involved in the virulence of several pathogens, including pathogenic mycoplasmas. In the protease repertoires identified in *M. hyopneumoniae* strains and *M. flocculare* samples, several quantitative differences were observed, which are suggestive of differential mechanisms for regulation of protein abundance and subcellular localization. Comparing proteases found in both *M. hyopneumoniae* 7448 and J SuPE, the oligoendopeptidase F and leucyl aminopeptidase were overrepresented in the pathogenic mycoplasma (MHP7448_0521, and MHP7448_0464, respectively). In comparison to *M. flocculare*, 6 proteases were overrepresented in *M. hyopneumoniae* 7448 samples, as follows. An aminopeptidase (MHP7448_0129) and the methionine aminopeptidase (MHP7448_0173) were more abundant in *M. hyopneumoniae* 7448 CyPE. The oligoendopeptidase F (MHP7448_0521), the leucyl aminopeptidase (MHP7448_0464), the ATP-dependent protease binding protein (MHP7448_0101), and the lon protease (MHP7448_0524) were more abundant in *M. hyopneumoniae* 7448 SuPE. Interestingly, the MHP7448_0129 aminopeptidase, overrepresented in *M. hyopneumoniae* 7448 CyPE, was differentially enriched in *M. flocculare*, being ~ 15 times more abundant in SuPE.

Redox balancing proteins can be also associated with virulence of several pathogens, including mycoplasmas, and some of them were differentially represented in the performed proteomic analyzes. *M. hyopneumoniae* neutrophil activating factor (MHP7448_0457) was detected only in *M. hyopneumoniae* 7448 CyPE. Regarding proteins differentially abundant, a thiol peroxidase (MHP7448_0096) and a thioredoxin (MHP7448_0384) (detected in CyPE and SuPE, respectively) were overrepresented in *M. hyopneumoniae* 7448 in comparison to *M. hyopneumoniae* J. In comparison to *M. flocculare*, a NADH oxidase (MHP7448_0082) and a thioredoxin reductase (MHP7448_0098) were overrepresented in the pathogenic mycoplasma.

Membrane transport proteins, such as ABC transporters, permeases and PTS system proteins, correspond to ~ 12% of the proteins encoded by *M. hyopneumoniae* and *M. flocculare* genomes. Around 47% (25) of membrane transporters species detected by LC-MS/MS were shared among the three analyzed proteomes. Among these membrane transporters, 6 (3 from CyPE and 3 from SuPE) and 3 (from SuPE) proteins were overrepresented and underrepresented, respectively, in *M. hyopneumoniae* 7448 in comparison to *M. hyopneumoniae* J. In comparison to *M. flocculare*, 7 (3 from CyPE and 4 from SuPE) and 1 (from CyPE) membrane transporters were overrepresented and underrepresented, respectively, in *M. hyopneumoniae* 7448.

Besides the canonical virulence-related proteins described above, *M. hyopneumoniae* 7448 also presents several potential virulence-related enzymes that were differentially represented in comparison to *M. hyopneumoniae* J, and *M. flocculare*. In comparison to *M. hyopneumoniae* J, a phosphopentomutase (MHP7448_0161), and a ribonuclease (MHP7448_0037) were 3–4 times more abundant in *M. hyopneumoniae* 7448 CyPE. Comparing to *M. flocculare*, a lipase-esterase (MHP7448_0133), a ribonuclease (MHP7448_0037), two glycolytic enzymes, namely lactate dehydrogenase (LDH, MHP7448_0137), and pyruvate dehydrogenase (represented by three of its four subunits: MHP7448_0116, MHP7448_0115, and MHP7448_0507), a chaperone DnaJ (MHP7448_0068), and three translation elongation factors (MHP7448_0075, MHP7448_0056 and MHP7448_0523) were from 2.6 to ~ 63 times more abundant in *M. hyopneumoniae* 7448.

Overall, these results showed important qualitative and quantitative differences in virulence-related proteins that might be PEP determinants. Importantly, along with these previously described virulence-related proteins, at least 47 other proteins were overrepresented in *M. hyopneumoniae* 7448 proteome in comparison to the samples of non-pathogenic mycoplasmas. The potential of these proteins as PEP determinants deserves further investigation.

### Differences between the protein repertoires of M. hyopneumoniae J and M. flocculare

The whole cell proteomes of *M. hyopneumoniae* J and *M. flocculare* were also qualitative and quantitatively analyzed and compared between each other. Among the proteins detected in *M. hyopneumoniae* J and *M. flocculare* samples, 26 and 41 proteins were exclusively detected, respectively (see Figure 1). Among the proteins shared between *M. hyopneumoniae* J and *M. flocculare*, 21 and 68 proteins from CyPE and SuPE, respectively, were differentially abundant (Supplementary Table 5C). The differences in abundance of both CyPE and SuPE proteins ranged from ~ 1.6 to ~ 19-times fold. Regarding CyPE differentially abundant proteins, 4 and 17 were overrepresented in *M. hyopneumoniae* J and *M. flocculare*, respectively. On the other hand, 63 and 5 SuPE proteins were overrepresented in *M. hyopneumoniae* J and *M. flocculare*, respectively. All 4 *M. hyopneumoniae* J overrepresented CyPE proteins were also overrepresented in SuPE samples. Interestingly, two *M. hyopneumoniae* J proteins, an ABC transporter (MHJ_0450) and an arginine-tRNA ligase (MHJ_0012), were differentially enriched, once they were underrepresented in CyPE and overrepresented in SuPE.

Overall, the comparisons between the proteomes of *M. hyopneumoniae* J and *M. flocculare* did not provide evidence of common features that could be clearly associated with the lack of virulence of these related bacteria. However, the observed qualitative and quantitative differences point out to physiological differences between them that deserve further investigation.

### Functional enrichment analyzes of the whole cell protein sets of M. hyopneumoniae 7448 and J, and M. flocculare

GO functional enrichment analyzes were performed for the whole cell protein sets of all mycoplasma samples to provide clues on functional differences between strains and species. Totals of 292 *M. hyopneumoniae* 7448 proteins (Supplementary Table 6A), 292 *M. hyopneumoniae* J proteins (Supplementary Table 6B), and 286 *M. flocculare* proteins (Supplementary Table 6C) were categorized according to GO terms into “biological process” (BP), “molecular function” (MF), and “cellular component”(CC) categories. No annotations were retrieved for 52, 51, and 29 proteins of *M. hyopneumoniae* 7448, *M. hyopneumoniae* J, and *M. flocculare*, respectively. Several functional BP, CC, and MF subcategories were commonly overrepresented in all mycoplasma samples. On the other hand, some functional subcategories were exclusively found as overrepresented in each of the analyzed samples, as follows. “Cellular macromolecule metabolic process”, “phosphorus metabolic process” and “ribose phosphate metabolic process” (BP subcategories); and “nucleic acid binding”, “oxidoreductase activity”, and “translation factor activity, RNA binding” (MF subcategories) were enriched only in *M. hyopneumoniae* 7448. For *M. hyopneumoniae* J. only the MF subcategories “hydrolase activity”, “nucleoside-triphosphatase activity”, and “pyrophosphatase activity” (MF) were exclusively enriched. Finally, some subcategories involved in nucleotide metabolism, as “pyridine nucleotide metabolic process”, “nucleobase-containing compound biosynthetic process”, “aromatic compound biosynthetic process” (BP subcategories), and other nucleotide-metabolism related MF subcategories were exclusively enriched in *M. flocculare*.

The performed GO functional analyzes showed that *M. hyopneumoniae* 7448 and J strains and *M. flocculare* present overall metabolic similarities, as expected. However, some interesting differences were highlighted among them, pointing out specific functional distinctions with possible impact for their proliferation, and survival capacities in the natural host.

### Uncharacterized proteins detected in the M. hyopneumoniae 7448 and J, and M. flocculare proteomes

**Figure 4.**
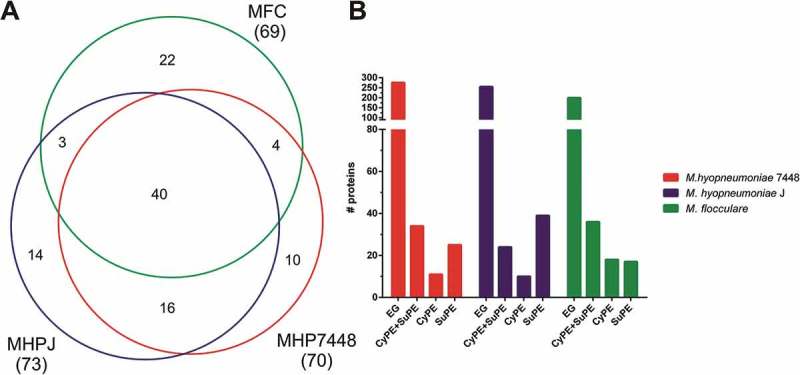
Overview of the uncharacterized proteins identified in *M. hyopneumoniae* 7448 and J, and *M. flocculare* samples. (A) Venn diagram of uncharacterized proteins detected in *M. hyopneumoniae* 7448 (MHP7448) and J (MHPJ), and *M. flocculare* (MFC) samples. Overall numbers of proteins identified for each sample between parentheses. The numbers of uncharacterized proteins exclusively detected in each sample or shared between them are indicated within the Venn diagram. (B) Distribution of uncharacterized proteins detected in CyPE and/or SuPE samples. Bar colors represent the different analyzed mycoplasma samples. CyPE+SuPE, proteins detected in both CyPE and SuPE samples; CyPE, proteins detected only in CyPE samples; SuPE, proteins detected only in SuPE samples. A bar representing the overall number of genes encoding uncharacterized proteins in the corresponding mycoplasma genome was included for reference (EG).

Our proteomic data provided experimental validation for 70, 73 and 69 genes previously regarded as hypothetical for *M. hyopneumoniae* 7448 and J, and *M. flocculare*, respectively, establishing interesting subsets of mycoplasma uncharacterized proteins (Supplementary Table 3), which deserve further attention. The Venn diagram in Figure 4(A), summarizes the exclusive and shared repertoires of detected *M. hyopneumoniae* 7448 and J, and *M. flocculare* uncharacterized proteins. Cell fraction analyzes revealed that ~ 44% of the uncharacterized protein species were found in both CyPE and SuPE (Figure 4(B)). A large number of uncharacterized protein species were exclusively detected in SuPE samples from *M. hyopneumoniae* 7448 (25, 35%) and J (39, 53%). For *M. flocculare*, only 24% of uncharacterized proteins were detected in SuPE.

*In silico* functional predictions were performed in order to provide clues on the functional roles of the detected uncharacterized proteins. A total of 41 different domains from the Pfam database were found distributed among 52 out of the total of 109 different uncharacterized proteins species detected in the analyzed samples (Supplementary Table 7). The “N-6 DNA methylase” domain was exclusively found in the *M. hyopneumoniae* 7448 set of uncharacterized proteins, while the DUF1410, ‘DUF4231ʹ and “tRNA synthetases class II” domains were exclusively found in the *M. hyopneumoniae* J set. Finally, domains related to replication initiation, peptidase and recombinase functions were found exclusively in the *M. flocculare* set.

Among the uncharacterized proteins shared between *M. hyopneumoniae* 7448 and J, and/or between *M. hyopneumoniae* 7448 and *M. flocculare*, 14 virulence-related domains were identified, including domains of potential peptidases, lipases, nucleases, permeases, thioredoxins and chaperones, were found distributed among 18 protein species. Among these proteins, only 4 were overrepresented in *M. hyopneumoniae* 7448, namely MHP7448_0431, bearing a “phosphatidylethanolamine-binding” domain; MHP7448_0064, bearing a “AAA domain”; MHP7448_0522, bearing a “GDSL-like lipase/acylhydrolase” domain; and MHP7448_0148, bearing a ‘Hsp33ʹ domain. While MHP7448_0431 and MHP7448_0064 were overrepresented in comparison to *M. hyopneumoniae* J, MHP7448_0431, MHP7448_0522 and MHP7448_0148 were overrepresented in comparison to *M. flocculare*.

## Discussion

Bacterial pathogenicity and virulence are multifactorial features that can be better assessed in comparative studies at the protein level, as protein abundance is the result of transcriptional regulation, post-translational processing and/or protein degradation. In this study, we compared the protein repertoires of cytoplasmic and surface-enriched protein fractions, comprehending the whole-cell proteomes, from the pathogenic and non-pathogenic *M. hyopneumoniae* strains (7448 and J, respectively), and *M. flocculare*, a non-pathogenic related species. For the first time, subcellular fractions of *M. hyopneumoniae* and *M. flocculare* were comparatively assessed using high-sensitivity high-resolution mass spectrometry. Qualitative and quantitative differences between the pathogenic *M. hyopneumoniae* 7448 and its non-pathogenic were found, involving potential PEP determinants, such as adhesins, proteases, and proteins related to redox balancing or membrane trafficking.

Cell fractioning procedures are useful to reduce proteome complexity, allowing the enrichment of low-abundance proteins. They improve the efficiency of MS-based protein identification and allow the association of different sets of proteins to specific cell compartments [17]. The carried out fractioning approach allowed to generate soluble fractions, enriched with cytoplasmic proteins (CyPE), and insoluble fractions, enriched with surface proteins (SuPE). For SuPE preparation, protein solubilization was carried out using the RapiGest SF surfactant, instead of the usual Triton X-114 or SDS solubilization protocols [18]. This surfactant allowed efficient protein solubilization in a one-step procedure and improved MS-protein identification. *In silico* subcellular localization prediction associated with the quantitative proteomics of CyPE and SuPE, confirmed their enrichment with cytoplasmic and surface proteins, respectively, for all analyzed mycoplasma samples. A previous *M. hyopneumoniae* 7448 surface protein survey carried out by our group identified only 34 surface-predicted proteins detected using a biotin cell surface labeling approach [19]. Our fractionation/solubilization approach, in turn, allowed the identification of 111 surface-predicted proteins in the *M. hyopneumoniae* 7448 SuPE (38% of the predicted surfaceome).

The cell fractioning approach combined with a high-resolution and sensitivity LC-MS/MS provided a high proteome coverage for all three mycoplasmas analyzed. The LC-MS/MS approach sensitivity was evidenced by comparing our data to those published by Pinto *et al*. (2009). In comparison to the former data, proteome coverage was improved 28% (from 22% to 50%) for *M. hyopneumoniae* 7448, and 27% (from 24% to 51%) for *M. hyopneumoniae* J. The remaining ~ 50% of predicted proteins not covered by our proteomic data may not have been detected due to their low abundance or lack of expression in culture conditions.

*M. hyopneumoniae* 7448 shared ~ 70% of the detected proteins with *M. hyopneumoniae* J and *M. flocculare*. Despite these high similarities between the sets of proteins detected for *M. hyopneumoniae* strains and *M. flocculare*, many qualitative and quantitative differences were detected, several of them likely associated with pathogenicity/PEP determination. Within the sets of proteins differentially represented in *M. hyopneumoniae* 7448 in comparison to the non-pathogenic samples, there are representatives of several classes of proteins and/or functions that may be potential PEP determinants, such as adhesins, proteases, oxidative stress-related proteins, and membrane transporters, among others.

Genomic comparative analyzes demonstrated that the sets of adhesin-encoding genes from *M. hyopneumoniae* 7448 and J, and *M. flocculare* are quite similar, containing few qualitative differences between the adhesin repertoires of *M. hyopneumoniae* and *M. flocculare* [9]. The only differences are the absence of *M. flocculare* orthologs for one P97 paralog (P97 copy-1, MHP7448_0198), and one P102 paralog (P102 copy-1, MHP7448_0199), and some rearrangements in *M. hyopneumoniae* genomic regions containing adhesin genes in comparison to *M. flocculare*. Despite these differences, the overall high qualitative similarity between the *M. hyopneumoniae* and *M. flocculare* adhesin sets was confirmed at proteomic level by the data described here. However, our data also pointed out some interesting quantitative differences, as three adhesins (P97-like, MgpA-like and P65) were more abundant in *M. hyopneumoniae* 7448 than in the non-pathogenic mycoplasmas, which may be associated with the higher adherence capacity of pathogenic *M. hyopneumoniae*. Conversely, four adhesins were more abundant in the non-pathogenic mycoplasmas. P95 and P97, for example, were more abundant in *M. flocculare* than in *M. hyopneumoniae* 7448. However, the *M. flocculare* orthologs are quite divergent (only ~ 55% of sequence identity to the *M. hyopneumoniae* orthologs), which may imply different adhesion properties. Moreover, *M. flocculare* has only one copy of P97 (MFC_00472), while *M. hyopneumoniae* has two, and, in this case, the overrepresentation of the single *M. flocculare* P97 may be resultant of a compensating mechanism.

Additionally, we observed higher peptide coverages in CyPE in comparison to those in SuPE, for *M. hyopneumoniae* and *M. flocculare* proteins, including adhesins. This suggests that these proteins are more fragmented in the cell surface than in in the cytoplasm, when they are expected to be mostly unprocessed. Previous studies have showed that adhesins are targets of post-translational proteolytic events [20–24] which can be differential between *M. hyopneumoniae* strains [15]. Along with differential adhesin abundance, the possibly differential adhesin post-translational proteolytic processing likely impact on bacterial pathogenicity and deserve further investigation.

As mediators of post-translational proteolytic events and other important cell processes, proteases play an important role to shape the *M. hyopneumoniae* proteome. Most of the proteases found in the whole cell proteomes of *M. hyopneumoniae* strains and *M. flocculare* were detected in both CyPE and SuPE. Interestingly, most of the overrepresented proteases of *M. hyopneumoniae* 7448 in comparison to *M. hyopneumoniae* J and *M. flocculare* were detected in SuPE. These differences in protease abundance between subcellular fractions and between pathogenic and non-pathogenic mycoplasmas could be resultant of differential enzyme activity or regulation for their targeting to preferential substrates in cell surface. With that, specific proteolytic activities could be targeted, for example, to the processing of surface adhesins.

Some of the proteases overrepresented in *M. hyopneumoniae* had their activities experimentally assessed [25–27]. Interestingly, *M. hyopneumoniae* leucyl aminopeptidase has been associated with plasminogen, heparin and foreign DNA binding and is localized on mycoplasma cell surface [26], which corroborated its higher abundance in *M. hyopneumoniae* 7448 SuPE. Moreover, oligoendopeptidase F and XAA-PRO aminopeptidase were previously associated with host kallikrein-kinin system, participating in inflammatory processes [27]. Overall, overrepresentation of proteases in the surface of *M. hyopneumoniae* 7448, along with previous functional studies, indicate the involvement of these enzymes with important pathogenicity-related mechanisms from adhesion to host immunomodulation.

Endogenous production of hydrogen peroxide through glycerol metabolism is essential for cytotoxicity of pathogenic mycoplasmas, as *Mycoplasma pneumoniae* and *Mycoplasma mycoides* subsp. *mycoides* [28,29]. In line with that, it was recently demonstrated that pathogenic strains of *M. hyopneumoniae* were able to produce hydrogen peroxide from glycerol metabolism, but that the non-pathogenic strain J and *M. flocculare* were not [11]. *M. hyopneumoniae* uptakes and metabolizes glycerol, while *M. flocculare* does not, failing to produce cytotoxic levels of hydrogen peroxide, which can be explained by the absence, in the *M. flocculare* genome, of the *glpO* gene, related to glycerol metabolism and hydrogen peroxide production [12].

Among proteins involved with oxidoreduction processes, a neutrophil activating factor was exclusively detected in *M. hyopneumoniae* 7448 CyPE. In *Helicobacter pylori*, this protein was previously related to neutrophil activation by the production of reactive oxygen species (ROS) [30]. Moreover, several redox balancing proteins were more abundant in *M. hyopneumoniae* 7448 than in *M. hyopneumoniae* J and *M. flocculare*. These results agreed with the functional enrichment analyzes, which demonstrated that the “oxidoreductase activity” subcategory, including all detected proteins related to redox balancing, was exclusively enriched in *M. hyopneumoniae* 7448. These differentially abundant proteins can be considered potential PEP determinants, due to their importance for bacterial survival in the context of endogenous (mycoplasma) and exogenous (host) ROS production [31–33]. For *M. flocculare*, its inability to produce endogenous hydrogen peroxide may be associated with its commensal nature, being less harmful to the host.

Membrane transporters have been described as virulence-related proteins, as they may be associated with multidrug resistance, metal ions uptake, and cell attachment [34], which are important for bacterial survival, and host colonization. *M. hyopneumoniae* and *M. flocculare* genomes have ~ 80 membrane transporters coding genes each, including genes coding for ABC transporters, permeases and PTS systems. In the LC-MS/MS analyzes, ~ 68% of the sets of membrane transporters species identified in *M. hyopneumoniae* and *M. flocculare* predicted proteomes were detected, with a partial (~ 50%) overlapping. Moreover, abundance differences were found between membrane transporters orthologs shared by *M. hyopneumoniae* 7448 and *M. hyopneumoniae* J, or by *M. hyopneumoniae* 7448 and *M. flocculare*. Overall, these evident qualitative and quantitative differences among the sets of membrane transporters of *M. hyopneumoniae* 7448, *M. hyopneumoniae* J, and *M. flocculare* are suggestive of substantial differences in transporting activities/capabilities and may also contribute to their differential virulence/pathogenicity.

Many proteins not classically related to virulence were also differential represented between *M. hyopneumoniae* 7448 and its non-pathogenic counterparts analyzed here. Functional enrichment analyzes showed some important metabolic subcategories specifically enriched in the *M. hyopneumoniae* 7448 whole cell proteome. The “phosphorous metabolic process” subcategory, which includes several glycolytic enzymes and kinases, and the “RNA binding” subcategory, which includes ribosomal proteins, translational elongation factors and aminoacyl tRNA ligases, were exclusively enriched in this pathogenic mycoplasma. In agreement to the functional enrichment analyzes, several proteins with canonical functions in metabolic pathways were overrepresented in *M. hyopneumoniae* 7448 protein repertoire, including the glycolytic enzymes LDH and pyruvate dehydrogenase, the pentose pathway enzyme phosphopentomutase, and translation-related proteins. Overall, the exclusive enrichment of all these metabolic functions suggests a higher metabolic capacity for the pathogenic *M. hyopneumoniae* strain, which may favor its proliferation and survival, contributing to the colonization, and infection of the porcine respiratory tract.

Glycolytic enzymes and other differential *M. hyopneumoniae* 7448 proteins not usually regarded as virulence factors, such as proteins involved in pentose phosphate pathway, DNA replication, and translation may have also alternative (moonlighting) functions of relevance for pathogenicity [35–37]. For instance, LDH is highly immunogenic and may have an immunomodulatory role [38,39], while pyruvate dehydrogenase and phosphopentomutase are proteins that play roles in adherence to the host extracellular matrix and DNA repair, respectively [40–42].

Around 37% of the sequenced genomes of *M. hyopneumoniae* strains and *M. flocculare* codes for hypothetical proteins. For pathogenic species, such set of hypothetical proteins is of particular interest, once it represents a potential reservoir of unknown virulence factors. For *M. pneumoniae*, many novel virulence factors were predicted upon *in silico* analyzes of hypothetical proteins [43]. In our study, several *M. hyopneumoniae* and *M. flocculare* coding DNA sequences (CDSs) whose putative products have been annotated as “hypothetical proteins” had their proteins products experimentally detected by LC-MS/MS. This allowed to confirm these CDSs as functional genes, and to change the status of their products to that of “uncharacterized proteins”. Among the detected uncharacterized proteins, several functional domains were predicted, including virulence-related ones, and most of them were conserved among the orthologs. More importantly, abundance differences between *M. hyopneumoniae* 7448 and its assessed non-pathogenic counterparts were observed for some of the uncharacterized proteins bearing functional domains, including virulence-related ones. Future analyzes of these and other uncharacterized proteins along with the characterization of their functional domains will be important steps towards the elucidation of their functions in *M. hyopneumoniae* biology and their possible roles as novel virulence factors.

## Conclusion

Our results provided a comprehensive profiling of the whole cell proteomes of two *M. hyopneumoniae* strains and *M. flocculare*, and an extended list of tens of candidates to pathogenicity determinants, beyond those classically described. Several protein classes with potential virulence-related functions were identified as overrepresented in the *M. hyopneumoniae* 7448 pathogenic strain, including adhesins, proteases, oxidative stress proteins, membrane transporters, and proteins with moonlighting functions, along with many so far uncharacterized proteins. Based on our proteomics results, the pathogenic nature of *M. hyopneumoniae* may be explained, at least in part, by the overrepresentation of several virulence-related proteins. These overrepresented proteins are involved in a wide range of biological processes, including adhesin processing and cell adhesion regulation, detoxification, overall metabolism regulation, and host-pathogen cell trafficking, among others. Although no specific commensalism determinants were found, the underrepresentation of several virulence-related proteins encoded by the non-pathogenic mycoplasmas may be a key point to explain their commensal natures.

Several of the identified proteins in *M. hyopneumoniae* strains and *M. flocculare* repertoires deserve future studies to elucidate mechanisms related to pathogenicity or commensalism, respectively. Of particular interest will be proteins with unknown function or with possible moonlighting functions overrepresented in the pathogenic *M. hyopneumoniae* 7448 strain. Moreover, the identification and characterization of *M. hyopneumoniae* virulence factors is of upmost relevance to discover new targets for the development of novel diagnostic methods, therapeutic drugs, and preventive vaccines against PEP.

## Materials and methods

### Bacterial growth conditions

*M. hyopneumoniae* pathogenic strain 7448 was isolated from an infected swine from Lindóia do Sul (SC, Brazil) [7]. *M. hyopneumoniae* non-pathogenic strain J (ATCC 25,934), and the non-pathogenic *M. flocculare* (ATCC 27,716) were acquired from American Type Culture Collection by the Empresa Brasileira de Pesquisa Agropecuária-Centro Nacional de Pesquisa de Suínos e Aves (EMBRAPA-CNPSA, Concórdia, SC, Brazil). For soluble and insoluble protein extracts, respectively, all bacteria were cultivated in 50 mL and 100 mL of Friis medium [44] for 48 h [45], at 37°C. Cultures were carried out independently in triplicates (biological replicates), and immediately used for protein extraction.

### Protein extraction and sample preparation for mass spectrometry

For protein extraction, cultured mycoplasma cells were pelleted by centrifugation (3500 x *g*, 15 min, 4°C), and washed three-times with PBS (pH 7.4). Cells were resuspended and lysed by sonication at 25 Hz in an ice bath by five 30 s cycles with 1 min intervals between pulses. The lysates were centrifuged at 10,000 x *g*, for 20 min, at 4°C and the supernatant (soluble fraction) was recovered for proteomics analyzes. The pellet (insoluble fraction) was resuspended in RapiGest SF Surfactant (Waters Corporation, Number 186,001,861). Soluble and insoluble protein extracts were quantified using the microBCA Protein Assay Kit (Thermo Fischer Scientific, Number 23,235) using a NanoDrop 2000 spectrophotometer (Thermo Fischer Scientific). Three protein extracts were independently produced to provide the three biological replicates for each sample.

Samples containing 100 μg and 50 μg of proteins from the soluble and insoluble fractions, respectively, were treated for MS analyzes. For soluble fraction analyzes, proteins were precipitated with TCA 20%-acetone, incubated for 16 h at 4°C, and further centrifuged at 20,000 × g for 10 min. Protein pellets were dried and then solubilized with 8 M urea. Next, proteins were reduced with 2 μg of DTT (Bio-Rad, Number 161–0611) at 37°C for 1 h, and alkylated with 10 μg of iodoacetamide (Bio-Rad, Number 163–2109) in the dark, at room temperature. Protein samples were diluted to a final 1 M urea concentration, and further digested with 1 μg of trypsin (Promega, Number V5280). For insoluble fraction analysis, samples resuspended in RapiGest SF were reduced with DTT (Bio-Rad) at 60°C for 30 min to a final concentration of 5 mM and alkylated with iodoacetamide (Bio-Rad) 15 mM (final concentration) at room temperature for 30 min in the dark. Proteins were then digested overnight with 0.5 μg of trypsin (Promega) at 37°C, and RapiGest SF was removed as recommended by the manufacturer (Waters). Resulting soluble and insoluble fractions peptides were desalted in HLB cartridges (Waters, Number 186,000,383), and eluted with 50% acetonitrile/0.1% TFA. Peptides were then lyophilized using a Concentrator Plus (Eppendorf), prior to MS analyzes.

### Mass spectrometry analyzes

Processed peptide samples were analyzed for protein identification using liquid chromatography-tandem mass spectrometry (LC-MS/MS) as described [16,46]. Briefly, each peptide sample was reconstituted using 0.1% formic acid in water, loaded onto a nanoAcquity HPLC system (Waters Corporation, MA, USA). A two-step LC was performed, using first a trap column PepMap 100 C18 LC column (300 µm x 5 mm) (Thermo Fischer Scientific, IL, USA), at a flow rate of 5 µl/min, and then an Easy-Spray Column PepMap RSLC C18 (75 µm x 15 cm) analytical column (Thermo Fischer Scientific). For the gradient elution, the mobile phase solvents consisted of 0.1% formic acid in water (solvent A), and 0.1% formic acid in acetonitrile (Burdick and Jackson) (solvent B). The gradient flow was set at 0.3 µl/min. The elution profile consisted of a hold at 5% solvent B for 5 min, followed by a ramp up to 35% solvent B over 25 min; a ramp up to 95% solvent B in 5 min; and a hold at 95% for 5 min, prior to a return to 5% solvent B in 5 min, and re-equilibration at 5% solvent B for 20 min. After LC, the peptides were introduced into a MS/MS Orbitrap Elite Hybrid Ion Trap-Orbitrap mass spectrometer (Thermo Fischer Scientific). A 2.0 kV voltage was applied to the nano-LC column. The mass spectrometer was programmed to perform data-dependent acquisition by scanning the mass-to-charge (m/z) range from 400 to 1600, at a nominal resolution setting of 60,000 for parent ion acquisition. For the MS/MS analyzes, the mass spectrometer was programmed to select the top 15 most intense ions with two or more charges. Each biological replicate was independently analyzed by LC-MS/MS two times (technical replicates).

### LC-MS/MS data analyzes

The MS/MS raw data were processed using msConvert version 3 (ProteoWizard) [47], and the peak lists were exported in the Mascot Generic Format (.mgf). MS/MS processed data were analyzed using Mascot Search Engine version 2.3.02 (Matrix Science, MA, USA) against local databases available for *M. hyopneumoniae* 7448 and J strains, and *M. flocculare*. These local databases were derived from the fully sequenced genomes from *M. hyopneumoniae* 7448 (920,079 bp), *M. hyopneumoniae* J (897,405 bp), and *M. flocculare* (763,948 bp), and included all deduced amino acid sequences (695, 672, and 581, respectively) from the corresponding genomes annotation (Siqueira et al 2013; Vasconcelos et al 2005), available at NCBI (https://www.ncbi.nlm.nih.gov/protein/) and Uniprot (http://www.uniprot.org/). The MASCOT search parameters for protein identification included a fragment ion mass tolerance of 0.5 Da, peptide ion tolerance of 7 ppm, and three missed cleavages of trypsin. Carbamidomethylation of cysteine was specified as a fixed modification, whereas the oxidation of methionine, acetylation of lysine and N-terminal ends of proteins, and phosphorylation of tyrosine and serine/threonine were specified as variable modifications [48].

Scaffold software version 4.8.1 (Proteome Software Inc., OR, USA) was used to validate the peptide and protein identifications. The peptide identifications were accepted if they could be established at greater than 99.0% probability as assigned by the Peptide Prophet algorithm [49]. The protein identifications were accepted if they could be established at greater than 95% probability as assigned by the Protein Prophet algorithm [50]; were based on at least 2 identified peptides; and were detected in at least two out of three replicates (both biological and technical).

### Identification of ortholog proteins among M. hyopneumoniae 7448 and J, and M. flocculare

In order to allow comparisons among protein repertoires from *M. hyopneumoniae* 7448 and J, and *M. flocculare*, ortholog sequences were determined using OrthoFinder [51]. Orthologs were then established based on the resulting bidirectional best hits, using as parameters identity ≥ 40% and a cutoff value of 1e^-6^.

### In silico subcellular localization predictions

Proteins identified in soluble and insoluble fractions of *M. hyopneumoniae* 7448 and J strains, and *M. flocculare* were analyzed *in silico* to predict their subcellular localization, being classified as surface or cytoplasmic proteins. Membrane proteins were initially predicted based on positive predictions as lipoproteins, using LipoP 1.0 [52], and PRED-LIPO [53]. Non-lipoproteins were then analyzed for transmembrane domain prediction using TMHMM v.2.0 [54], Phobius [55], HMMTOP [56], CW-PRED [57], and HMM-TM [58]. Non-transmembrane proteins were further analyzed for subcellular localization using PSORTb v. 3.0.2 [59], iLoc-Gpos [60], and CELLO v.2.5 [61].

Proteins not predicted as membrane proteins were then classified as secreted or cytoplasmic. Secreted proteins were predicted based on the presence of signal peptide or on non-classical secretion prediction. Signal peptide predictions were made using SignalP 4.1 [62], Phobius [55], and PrediSi [63]. Non-classical secretion was predicted using SecretomeP 1.0 [64]. Remaining proteins, not classified as membrane or secreted proteins, were considered cytoplasmic proteins. For any given prediction, coincidence in all or at least most of the used predictors was required for validation.

### Quantitative and qualitative comparisons between LC-MS/MS data of insoluble and soluble protein extracts from M. hyopneumoniae 7448 and J, and M. flocculare

To confirm the enrichment of surface proteins in the insoluble fractions, the LC-MS/MS datasets of proteins identified in the *M. hyopneumoniae* 7448 and J, and *M. flocculare* insoluble fractions were compared to those of the corresponding soluble fractions. For that, differentially represented proteins, exclusively detected or more abundant in the insoluble protein fraction in comparison to the soluble extract of the same species or strain, were analyzed based on subcellular localization predictions. Protein abundance was measured based on normalized spectral abundance factor (NSAF) values [65] and quantitative differences between proteins detected in both insoluble and soluble protein fractions were statistically analyzed in Scaffold software using the Student’s *t*-test, with the Benjamini-Hochberg FDR multiple-testing correction. A *p*-value < 0.05 was considered statistically significant. Proteins with differential abundances between surface-enriched and soluble protein extracts were represented in heat-maps using the Heatmapper web server (http://www.heatmapper.ca) using the Z-score calculation of NSAF values.

### Comparative quantitative analyzes of proteins shared between M. hyopneumoniae 7448, J, and M. flocculare

For quantitative comparisons between ortholog proteins shared between (i) *M. hyopneumoniae* 7448 and *M. hyopneumoniae* J; (ii) *M. hyopneumoniae* 7448 and *M. flocculare*; and (iii) *M. hyopneumoniae* J and *M. flocculare*, the analyzes were based on the exponentially modified protein abundance index (emPAI) values [66]. EmPAI values were calculated for each protein in the Scaffold software, not using the normalization option, to allow intraprotein (between ortholog proteins), and intersample (*M. hyopneumoniae* 7448 vs. *M. hyopneumoniae* J; *M. hyopneumoniae* 7448 vs. *M. flocculare*; or *M. hyopneumoniae* J vs. *M. flocculare*) comparisons. The emPAI values were statistically compared using Student’s *t*-test using Prism GraphPad Software version 6 (GraphPad Software, Inc, CA, USA). Fold-changes (FC) were calculated for each pair of ortholog proteins. Proteins with a *p*-value < 0.05 and a FC > 1.5 were considered differentially abundant by both statistical and FC parameters.

### In silico functional analyzes

*In silico* functional analyzes of *M. hyopneumoniae* and *M. flocculare* proteins identified by LC-MS/MS were based on gene ontology (GO). Mycoplasma identified proteins were submitted to hierarchical GO overrepresentation tests using the Cytoscape 2.6.3 26 plugin BiNGO 2.3 [67]. Custom *M. hyopneumoniae* 7448 and J GO annotation files were acquired from Uniprot (http://www.uniprot.org/). *M. flocculare* GO annotations were acquired using BLAST2GO version 3.0 [68]. For that, online BlastP searches were performed against the NCBInr database and GO mapping, and annotation were performed based on BlastP results (E-value ≤ 1.0 × 10^−3^). The ontology files were retrieved from the GO database (http://www.geneontology.org/). Both annotation and ontology files were edited in-house as BiNGO input files. The hypergeometric overrepresentation tests were performed at a 0.05 level of significance, with the Benjamini-Hochberg FDR multiple-testing correction. Uncharacterized proteins were further analyzed in order to predict functional domains using the Pfam software version 29.0 (http://pfam.xfam.org/) [69].
